# Neuroprotective Potential of *Caulerpa lentillifera* Extracts against Lipopolysaccharide‐Induced Neuroinflammation In Vitro

**DOI:** 10.1002/alz70859_107706

**Published:** 2025-12-25

**Authors:** Aiman Hamli, Kalavathy Ramasamy, Snezana Agatonovic‐Kustrin, Syed Adnan Ali Shah, Nur Syafiqah Rahim, Siong Meng Lim

**Affiliations:** ^1^ Universiti Teknologi MARA (UiTM), Bandar Puncak Alam, Selangor Malaysia; ^2^ Collaborative Drug Discovery Research (CDDR) Group, Faculty of Pharmacy, Universiti Teknologi MARA (UiTM), Puncak Alam, Selangor Malaysia; ^3^ Faculty of Pharmacy, Universiti Teknologi MARA, Puncak Alam, Selangor Malaysia; ^4^ Sechenov First Moscow StateMedical University (Sechenov University), Bolshaya Pirogovskaya, Moscow Russian Federation; ^5^ La Trobe University, Bendigo, VIC Australia; ^6^ Universiti Teknologi MARA (UiTM), Arau, Perlis Malaysia; ^7^ Universiti Teknologi MARA (UiTM), Puncak Alam, Selangor Malaysia

## Abstract

**Background:**

Current pharmacological therapies against Alzheimer’s disease (AD) merely relieve the clinical symptoms but fail to halt the progression of the neurodegenerative disease. This raises the need for alternative neuroprotective agents that can prevent AD. Based on its antioxidant and anti‐inflammation properties, the marine alga, *Caulerpa lentillifera*, appears to be useful against the neuroinflammation‐driven AD. As such, this study aimed at uncovering the neuroprotective potential of *C. lentillifera* extracts against lipopolysaccharide (LPS)‐induced neuroinflammation in vitro.

**Method:**

Briefly, freeze‐dried *C. lentillifera* was extracted using methanol, and partitioned into hexane, chloroform and ethyl acetate. The resultant *C. lentillifera* extracts [i.e., methanol (CLME], hexane (CLHE), chloroform (CLCE), ethyl acetate (CLEAE)] were evaluated for antioxidant activity (DPPH) and COX‐1 inhibition using high‐performance thin layer chromatography (HPTLC). The extracts were also tested against LPS‐challenged macrophage (RAW 264.7) and neuroblastoma (SK‐N‐SH) cells. Reactive oxygen species (ROS) production, nitric oxide (NO) levels and caspase‐3 activity were measured. Phytochemical profiling was performed using nuclear magnetic resonance (NMR) spectroscopy.

**Result:**

HPTLC found CLCE to exhibit the highest DPPH scavenging activity but relatively weak COX‐1 inhibition. When compared to CLCE, CLHE elicited moderate DPPH scavenging activity but relatively stronger COX‐1 inhibition whilst CLEAE exhibited the greatest COX‐1 inhibition. For RAW 264.7 cells, CLCE and CLHE were the two most potent extracts that significantly reduced ROS (≥ ‐36.24%), NO (≥ ‐43.71%) and caspase‐3 activity (≥ ‐95.26%) in LPS‐challenged cells. For SK‐N‐SH cells, CLCE and CLHE were also the two most potent extracts that significantly reduced ROS (≥ ‐71.73 %) and Caspase‐3 activity (≥ ‐54.67%) in LPS‐challenged cells. Phytochemical profiling of the extracts found CLCE to be presented with the highest phenolic content, followed by CLHE, CLME and CLEAE. NMR spectroscopy analysis revealed that CLME contained sugar and sterols; CLCE contained palmitic acids, polysaccharides and amino acids; CLEAE contained polysaccharides, terpenoids and phenolic compounds; CLHE contained fatty acids and oleic acids.

**Conclusion:**

*C. lentillifera* extracts, particularly CLME and CLHE, could potentially confer neuroprotection against neuroinflammation by reducing oxidative stress, inflammation, and apoptosis.

**Funding**: Fundamental Research Grant Scheme, Ministry of Higher Education, Malaysia (FRGS/1/2019/STG03/UITM/03/1).

